# Evaluation of microbial globin promoters for oxygen-limited processes using *Escherichia coli*

**DOI:** 10.1186/s13036-017-0082-3

**Published:** 2017-11-13

**Authors:** Alvaro R. Lara, Karim E. Jaén, Juan-Carlos Sigala, Lars Regestein, Jochen Büchs

**Affiliations:** 1 Departamento de Procesos y Tecnología, Universidad Autónoma Metropolitana-Cuajimalpa, Av. Vasco de Quiroga 4871, Santa Fe, C.P., 05348 Mexico City, Mexico; 2 RWTH Aachen University, AVT - Biochemical Engineering, NPG2 Forckenbeckstrasse 51, 52074 Aachen, Germany

**Keywords:** Microaerobic promoters, Oxygen-limited cultures, Globin promoters, FbFP expression, Microbioreactors

## Abstract

Oxygen-responsive promoters can be useful for synthetic biology applications, however, information on their characteristics is still limited. Here, we characterized a group of heterologous microaerobic globin promoters in *Escherichia coli*. Globin promoters from *Bacillus subtilis*, *Campylobacter jejuni*, *Deinococcus radiodurans*, *Streptomyces coelicolor*, *Salmonella typhi* and *Vitreoscilla stercoraria* were used to express the FMN-binding fluorescent protein (FbFP), which is a non-oxygen dependent marker. FbFP fluorescence was monitored online in cultures at maximum oxygen transfer capacities (OTR_max_) of 7 and 11 mmol L^−1^ h^−1^. Different FbFP fluorescence intensities were observed and the OTR_max_ affected the induction level and specific fluorescence emission rate (the product of the specific fluorescence intensity multiplied by the specific growth rate) of all promoters. The promoter from *S. typhi* displayed the highest fluorescence emission yields (the quotient of the fluorescence intensity divided by the scattered light intensity at every time-point) and rate, and together with the promoters from *D. radiodurans* and *S. coelicolor*, the highest induction ratios. These results show the potential of diverse heterologous globin promoters for oxygen-limited processes using *E. coli*.

**Electronic supplementary material:**

The online version of this article (10.1186/s13036-017-0082-3) contains supplementary material, which is available to authorized users.

## Introduction

Oxygen limitation can easily occur in high cell-density cultures due to technical and economic constraints that limit mass transfer in bioreactors. Oxygen limitation is commonly undesirable in cultures of *E. coli* because it causes strong unwanted metabolic deviations. However, operating the bioreactor at maximum oxygen transfer capacities (OTR_max_) would be advantageous from an economy standpoint. By modifying the metabolism of *E. coli*, it is possible to decrease the amount of byproducts formation and to improve the biomass yield and growth rate under microaerobic conditions [1]. Consequently, oxygen-limited bioprocesses could be an interesting option for the synthesis of valuable molecules, using self-inducible promoters that trigger transcription upon oxygen limitation. The development of such processes will require the availability of characterized promoters for the assembly of synthetic pathways. Oxygen-responsive promoters could also be applied as biosensors to detect oxygen-limited zones in bioreactors. We have previously characterized the performance of homologous oxygen sensitive promoters of *E. coli* and the promoter of the *Vitreoscilla stercoraria* hemoglobin (P_*vgb*_) in oxygen-limited cultures [2]. From a group of 14 promoters evaluated, P_*vgb*_ showed interesting characteristics like good repression under aerobic conditions and the highest induction ratio. This suggests that heterologous globin promoters could be viable tools for driving oxygen responsive gene expression in *E. coli*. Koskenkorva and coworkers [3] searched globin promoters from *Bacillus subtilis* (P_*Bs*_), *Campylobacter jejuni* (P_*Cj*_), *Deinococcus radiodurans* (P_*Dr*_), *Streptomyces coelicolor* (P_*Sc*_), and *Salmonella typhi* (P_*St*_). The promoters were isolated and cloned in a plasmid to express chloramphenicol acetyl transferase (CAT) in *E. coli*. When cultured in shake flasks at low shaking frequency (150 rpm), maximum CAT activity was reported for all promoters after 2 h of culture, and decreased afterwards [3]. Despite the relevance of such results, further characterization of the promoters under defined conditions is required. Namely, the cultures were performed in complex medium without pH and dissolved oxygen tension (DOT) monitoring. Furthermore, the OTR was not reported, and the dynamics of CAT expression in cultures not shown. Synthetic biology applications require standardized and well characterized parts. In this context, the effect of environmental conditions on the promoter activity is of prime relevance, particularly if bioprocess applications are sought. In the present contribution, the abovementioned promoters were synthesized and used to express the FMN binding fluorescent protein (FbFP). FbFP is an adequate reporter because of its fast activation independent from oxygen [4]. The assembly included the Shine-Dalgarno sequence and 8 bp spacer region as in our previous report [2], which allows a direct comparison of the results. Oxygen-limited cultures were performed in round well microtiter plates with optodes for pH and DOT monitoring using a chemically defined medium. Two filling volumes (1500 and 2400 μL per well) were used, which result in OTR_max_ values of ca. 11 and 7 mmol L^−1^ h^−1^, respectively [5]. Expression of FbFP under control of the constitutive promoter P_*kat*_ (which controls the expression of the aminoglycoside phosphotransferase gene *kat*), was used as a control to assess the effect of OTR_max_ in a constitutive expression system.

## Results and discussion

**Fig. 1:**
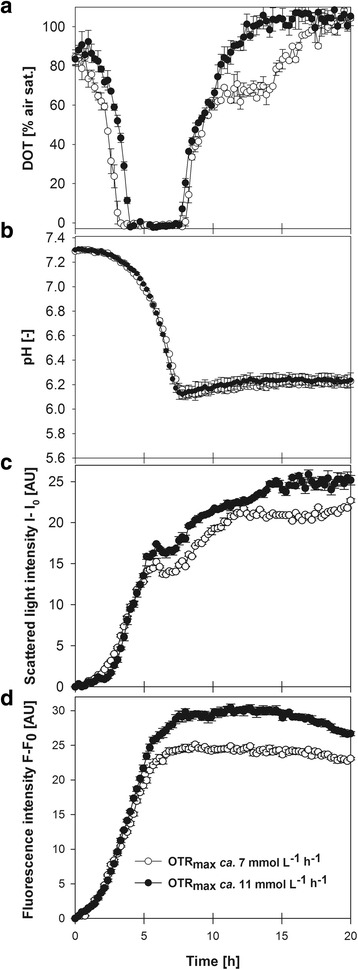
Oxygen-limited cultures of *E. coli* expressing FbFP under control of P_*kat*_. Online monitoring of DOT (**a**), pH (**b**), cell growth by scattered light (**c**) and FbFP fluorescence data are shown (**d**). Culture conditions: 48-well round well plate, *n* = 700 rpm, d_0_ = 3 mm, V_L_ = 1500 (for OTR_max_ ca. 11 mmol L^−1^ h^−1^) or 2400 μL (for OTR_max_ ca. 7 mmol L^−1^ h^−1^), mineral medium buffered with MOPS (0.2 M) plus 5 g/L of glucose. Vertical lines show the standard deviation (*n* = 3) of average values

Figure 1 shows the growth profiles of cultures expressing FbFP under control of P_*kat*_. Cultures were oxygen-limited after 3 and 4 h of inoculation and lasted for 3.5 and 4 h at OTR_max_ ca. 7 and 11 mmol L^−1^ h^−1^, respectively (Fig. 1a). The pH decreased until glucose exhaustion (indicated by a sudden increase of DOT) and slightly increased thereafter, presumably due to the consumption of acid species like fermentative byproducts (Fig. 1a, b). Cell growth monitored by scattered light showed a change of trend when oxygen limitation started and ceased when DOT reached saturation (Fig. 1a, c). The FbFP fluorescence signal increased in parallel to scattered light. Both, final biomass and FbFP fluorescence were higher at OTR_max_ ca. 11 mmol L^−1^ h^−1^ than at OTR_max_ ca. 7 mmol L^−1^ h^−1^ (Fig. 1c, d). This can be attributed to the metabolic adaptations of *E. coli* when oxygen limits energy generation. The lower OTR_max_ caused a decrease of approximately 20% on the biomass and FbFP fluorescence attained (Fig. 1c, d).

**Fig. 2:**
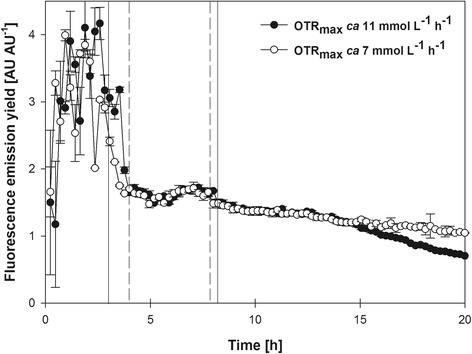
Fluorescence emission yield during oxygen-limited cultures of *E. coli* expressing FbFP under control of P_*kat*_. The vertical solid (for OTR_max_ ca. 7 mmol L^−1^ h^−1^) and dashed (for OTR_max_ ca. 11 mmol L^−1^ h^−1^) lines indicate the beginning (lines to the left) and end (lines to the right) of the oxygen-limited phases. Fluorescence yields were calculated by dividing the FbFP fluorescence intensity signal by the scattered light intensity signal at every time-point. Vertical lines show the standard deviation (*n* = 3) of average values

The fluorescence emission yields (calculated by dividing the FbFP fluorescence intensity signal by the scattered light intensity signal at every time-point) relate the FbFP fluorescence intensity with the biomass concentration. As shown in Fig. 2, the fluorescence emission yields under both OTR_max_ conditions were relatively high during the aerobic phase, although displaying a strong variation. During the oxygen-limited phase, the fluorescence yields rapidly dropped to a relatively stable value near to 1.5 AU AU^−1^. This suggests that the activity of the P_*kat*_ is affected by oxygen-limited conditions at the same extent than general biosynthetic capacity.

**Fig. 3:**
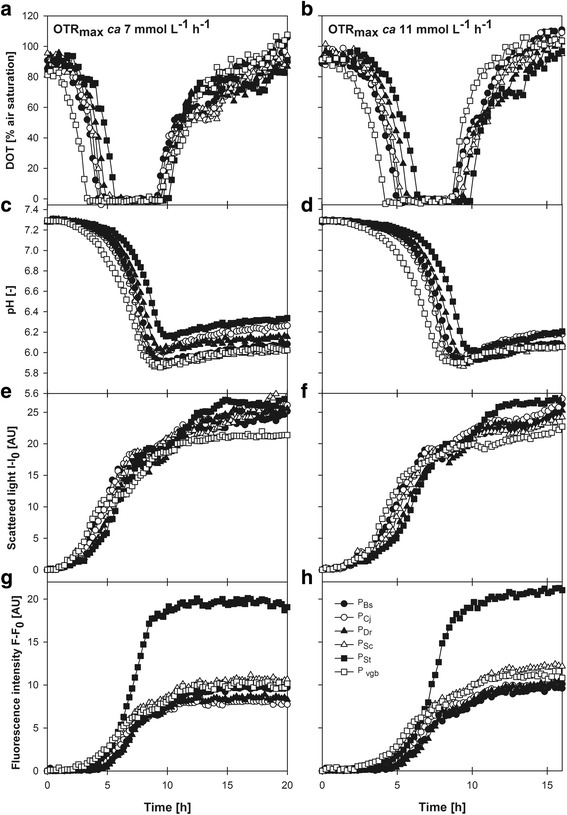
Oxygen-limited cultures of *E. coli* expressing FbFP under control of the globin promoters. Online monitoring of DOT (**a**, **b**), pH (**c**, **d**), cell growth by scattered light (**e**, **f**) and FbFP fluorescence data are shown (**g**, **h**). Culture conditions: 48-well round well plate, *n* = 700 rpm, d_0_ = 3 mm, V_L_ = 1500 (for OTR_max_ ca. 11 mmol L^−1^ h^−1^) or 2400 μL (for OTR_max_ ca. 7 mmol L^−1^ h^−1^), mineral medium buffered with MOPS (0.2 M) plus 5 g/L of glucose. Data from single experiments are shown for clarity

The growth profiles of the strains bearing the microbial globin promoters are shown in Fig. 3. Oxygen-limited cultures at two OTR_max_ (ca. 7 and 11 mmol L^−1^ h^−1^) were also performed to evaluate the sensitivity of the promoters to oxygen availability, which is very informative for bioreactor operation. A more restricted oxygen supply (resulting from a lower OTR_max_) may mimic the effect of a higher concentration of a chemical inducer (for instance, IPTG in the case of P_*lac*_). However, under oxygen-limited conditions, energy generation is also limited by the capacity to regenerate NADH, which is also reflected in the capacity for biomass synthesis. In cultures at OTR_max_ ca. 7 mmol L^−1^ h^−1^, oxygen was depleted between 3 and 5 h after inoculation (Fig. 3a). Similar to culture profiles of Fig. 1, the pH decreased during the cultures until the raise of DOT signal, indicative of glucose exhaustion (Fig. 3c). The attained biomass was different for the strains bearing the different promoters and ranged from 22 (for P_*vgb*_) to 28 (for P_*St*_) AU (Fig. 3e). The FbFP fluorescence signals were very low during the first 4 h and increased importantly thereafter, coincident with the period of oxygen limitation (Fig. 3g). The highest FbFP fluorescence signal was recorded for P_*St*_, which reached nearly 20 AU. Although this value is similar of that obtained using P_*kat*_, the increase of fluorescence was observed only during the oxygen-limited period for P_*St*_. The FbFP fluorescence readings for strains bearing P_*Bs*_, P_*Dr*_, P_*Sc*_ and P_*vgb*_ were similar, while that of the culture using P_*Cj*_ was the lowest of all the studied promoters, attaining only 8 AU (Fig. 3g). In cultures at OTR_max_ ca. 11 mmol L^−1^ h^−1^, oxygen was depleted between 4 and 6 h after inoculation (Fig. 3b). The pH and DOT profiles were similar to those in cultures at OTR_max_ ca. 7 mmol L^−1^ h^−1^ (Fig. 3b, d). In contrast to cultures using P_*kat*_, the biomass concentrations reached using the different globin promoters at OTR_max_ ca. 11 mmol L^−1^ h^−1^ were only slightly higher than those at OTR_max_ ca. 7 mmol L^−1^ h^−1^ for P_*Bs*_, P_*Cj*_, and P_*vgb*_ while slightly decreased for P_*St*_ and remained nearly the same for P_*Dr*_ and P_*Sc*_ (Fig. 3e and f). In cultures at OTR_max_ ca. 11 mmol L^−1^ h^−1^ the FbFP fluorescence increased to a small extent for P_*Cj*_, P_*Sc*_, and P_*St*_, while remained unchanged for P_*vgb*_ and even decreased for P_*Bs*_ and P_*Dr*_.

**Fig. 4:**
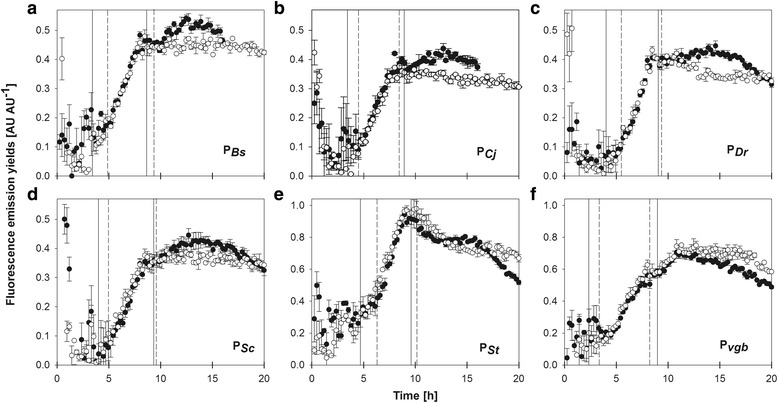
Fluorescence emission yields during oxygen-limited cultures of *E. coli* expressing FbFP under control of the globin promoters. The vertical solid (for OTR_max_ ca. 7 mmol L^−1^ h^−1^) and dashed (for OTR_max_ ca. 11 mmol L^−1^ h^−1^) lines indicate the beginning (left lines) and end (right lines) of the oxygen-limited phases. Fluorescence yields were calculated by dividing the FbFP fluorescence intensity signal by the scattered light intensity signal at every time-point. Empty symbols: cultures at OTRmax ca. 7 mmol L^−1^ h^−1^. Filled symbols: cultures at OTRmax ca. 11 mmol L^−1^ h^−1^. Note that the scales on the Y axes are changing. Vertical lines show the standard deviation (*n* = 3) of average values. **a**: P_*Bs*_; **b**: P_*Cj*_; **c**: P_*Dr*_; **d**: P_*Sc*_; **e**: P_*St*_; **f**: P_*vgb*_

Figure 4 shows the fluorescence emission yields through the cultures of the strains bearing the globin promoters. As can be seen for all the globin promoters, the fluorescence emission yields were low during the aerobic phase of the cultures. In this phase the fluorescence emission yields were disperse, which may be a result of a certain degree of induction during the pre-culture development. Shortly after oxygen limitation, the fluorescence yields started to increase, indicating a fast induction of the *fbfp* gene (Fig. 4). A fast induction of FbFP expression was also observed under the control of promoters from fermentative pathways of *E. coli* [2]*.* Those promoters are activated by the protein FNR (fumarate nitrate reduction), which senses oxygen activating transcription through a redox reaction. It has been demonstrated that P_*vgb*_ [6] and P_*Bs*_ [7] are also activated by FNR. Koskenkorva and coworkers found FNR binding sites sequences in P_*Cj*_ and P_*St*_, but not in P_*Dr*_ and P_*Sc*_ [3]. The globins of *C. jejuni* and *S. typhi* are expressed in response to stress by nitric oxide, however, the role of FNR on the regulation of these promoters under oxygen-limited conditions is not completely defined [8, 9]. Nevertheless, from Fig. 4 it can be seen that all the globin promoters studied can efficiently trigger the expression of FbFP upon oxygen limitation in *E. coli*. In cultures at OTR_max_ ca. 7 mmol L^−1^ h^−1^, the FbFP fluorescence remained relatively constant after oxygen limitation (Fig. 4 g). In contrast, in cultures at OTR_max_ ca. 11 mmol L^−1^ h^−1^, the FbFP fluorescence increased slightly for the different promoters after oxygen limitation. This indicates that some FbFP can be synthesized from re-assimilation of fermentative by-products (which are produced by *E. coli* under oxygen limitation) in cultures at OTR_max_ ca. 11 mmol L^−1^ h^−1^, but not at the lower OTR_max_.

During the oxygen-limited period the fluorescence yields were similar for cultures at OTR_max_ of 7 or 11 mmol L^−1^ h^−1^ for all the promoters. The fluorescence yields for P_*St*_ and P_*vgb*_ were noticeably higher than for the rest of the globin promoters. These results differ from the previous report from Koskenkorva and coworkers [3], who found that the P_*Dr*_ displayed the highest activity. These differences could be related to genetic factors and culture conditions. First, RBS used in this work and the reported by Koskenkorva and coworkers [3] are different. Also, the use of different 5′ UTR sequences and/or reporter genes as compared with these authors could lead to differences in regulation or apparent promoter strength through unwanted interactions on different levels of expression [10, 11]. Concerning the culture conditions, the studies by Koskenkorva et al. [3] were performed using LB medium, and an *E. coli* K12 strain, which could produce different results. Moreover, cultures were carried out in unbuffered medium [3], and therefore strong pH fluctuations are expected [12]. However, pH values were not informed by the authors. In the present study, the maximum fluorescence emission yields were reached during the phase of DOT raise. The maximum fluorescence emission yield was greater in cultures at OTR_max_ ca. 11 mmol L^−1^ h^−1^ than in cultures at OTR_max_ ca. 7 mmol L^−1^ h^−1^ for most promoters, except for P_*St*_ and P_*vgb*_. In all cases, the fluorescence yield were relatively stable after oxygen raise when the OTR_max_ was ca. 7 mmol L^−1^ h^−1^, but rapidly decreased at OTR_max_ ca. 11 mmol L^−1^ h^−1^. Again, P_*St*_ and P_*vgb*_ were the exceptions, as fluorescence yields decreased fast after the point of DOT raise (Fig. 4e and f).

**Fig. 5:**
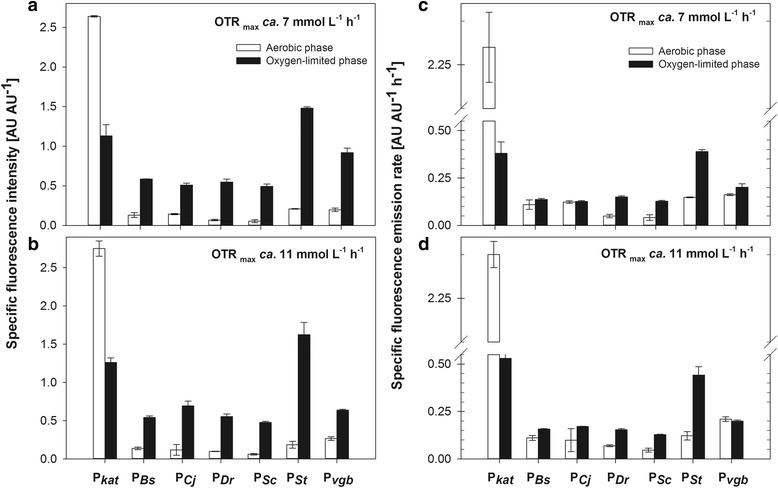
Specific fluorescence intensity (**a**, **b**) and specific fluorescence emission rate (**c**, **d**) during the oxygen-limited cultures. Specific fluorescence intensity was calculated as the slope in the plot of FbFP fluorescence intensity (F-F_0_) vs scattered light intensity (I-I_0_). Specific fluorescence emission rate was calculated multiplying the specific growth rate of the corresponding time period by the specific fluorescence emission. The OTR_max_ of the cultures is indicated in each graphic. White bars represent the values corresponding to the aerobic phase and black bars those corresponding to the oxygen-limited phase. Values are average of three cultures. Vertical lines show the standard deviation (*n* = 3) of average values

The characterization of promoters should also consider factors like growth rate to provide information about the impact of the expression of the gene of interest on the general metabolic activity. The specific fluorescence emission rate involves the specific growth rate (not shown) during the time period of the calculation. Therefore, it is useful to give an insight of the production rate of a protein of interest under control of the promoter used. The specific fluorescence intensity was calculated over the aerobic and oxygen-limited phases of the cultures and depicted in Fig. 5a and b. The specific fluorescence intensity was very low for all promoters during the aerobic phase of the cultures and increased substantially under oxygen-limited conditions in close agreement with data from Fig. 4. In cultures at OTR_max_ ca. 7 mmol L^−1^ h^−1^, the highest specific fluorescence intensity was observed for P_*St*_ (1.48 ± 0.02 AU AU^−1^) and P_*vgb*_ (0.92 ± 0.06 AU AU^−1^) (Fig. 5a). In cultures at OTR_max_ ca. 11 mmol L^−1^ h^−1^, most of the specific fluorescence intensity values were higher than those at OTR_max_ ca. 7 mmol L^−1^ h^−1^ (*p < 0.05 was evaluated and significant difference confirmed), except for P_*vgb*_, that reached 0.64 ± 0.01 AU AU^−1^ (Fig. 5b). Therefore, it can be concluded that stronger oxygen limitation resulted in stronger induction of P_*vgb*_ and not for the other promoters. In cultures at OTR_max_ ca. 11 mmol L^−1^ h^−1^, the highest specific fluorescence intensity was displayed again by P_*St*_, followed by P_*Cj*_, that reached values of 1.62 ± 0.10 and 0.69 ± 0.06 AU AU^−1^, respectively (Fig. 5b). These values are in general greater than those of endogenous promoters of *E. coli* under similar culture conditions [2]. The specific fluorescence emission rate was similar for P_*Bs*_ and P_*Cj*_ during the aerobic and oxygen-limited phase of cultures at OTR_max_ ca. 7 mmol L^−1^ h^−1^ (Fig. 5c). This means that despite the increase of fluorescence intensity observed upon oxygen depletion for these promoters, the decline of growth rate was more pronounced, resulting in a nearly unchanged fluorescence emission rate. For all the other promoters, the specific fluorescence emission rates increased during the oxygen-limited phase, compared to the aerobic phase of the culture at OTR_max_ ca. 7 mmol L^−1^ h^−1^ (Fig. 5c).

The highest specific fluorescence emission rate under oxygen-limited conditions was observed for P_*St*_ (Fig. 5c). In cultures at OTR_max_ ca. 11 mmol L^−1^ h^−1^ the specific fluorescence emission rate increased during the oxygen-limited phase, compared to the aerobic phase for the different promoters, except for P_*vgb*_ (Fig. 5d). The result for P_*vgb*_ is coincident with a previous study under similar conditions [2]. The specific fluorescence emission rates under oxygen-limited conditions were greater at OTR_max_ ca. 11 mmol L^−1^ h^−1^, compared to those obtained at OTR_max_ ca. 7 mmol L^−1^ h^−1^. This is most probably a result of the limited resources for energy generation and biomass synthesis under oxygen-limitation.

**Fig. 6:**
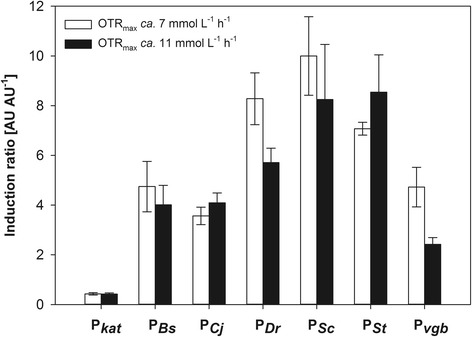
Induction ratio of the globin promoters characterized. The induction ratio was calculated dividing the value of the specific fluorescence intensity during the aerobic phase by the corresponding value during the oxygen-limited phase. White bars correspond to cultures at OTR_max_ ca. 7 mmol L^−1^ h^−1^ and black bars to cultures at OTR_max_ ca. 11 mmol L^−1^ h^−1^. Values are average of three cultures. Vertical lines indicate the standard deviation (*n* = 3) of average values

Figure 6 depicts the induction ratio. This parameter represents the change of specific fluorescence intensity under uninduced (aerobic) and induced (oxygen-limited) conditions in the cultures at different OTR_max_. The induction ratio was greater at OTR_max_ ca. 7 mmol L^−1^ h^−1^ for P_*Dr*_ and P_*vgb*_, while for the other promoters no significant differences were found using a T-test (*p* < 0.05). While P_*St*_ produced the greatest fluorescence intensity and fluorescence emission rate, expression under control of P_*Sc*_ was better repressed under aerobic conditions and yielded the highest induction ratio. The induction ratio of all the globin promoters was greater than the reported for endogenous promoters [2]. Although the used promoters, except P_*Sc*_ and P_*Dr*_ have putative regions for regulation by CRP, ArcA and FNR, the positions of these transcriptional elements are different for each promoter [3] and from the typical positions in *E. coli* [13]. It is possible then that the exact architecture and binding sequences of the heterologous promoters drive a more efficient induction under oxygen-limited conditions than the homologous promoters reported elsewhere [2].

The data set presented here provides useful information for the selection of oxygen sensitive promoters for particular designs. Severe oxygen limitation (OTR_max_ ca. 7 mmol L^−1^ h^−1^) seems to negatively affect the activity of most of the globin promoters studied. Nevertheless, cell engineering strategies aimed at improving the metabolic performance and energy generation by aerobic respiration of *E. coli* under oxygen-limited conditions can increase the specific fluorescence emission rate [2]. Altogether, the information shown contributes to expand the toolbox for synthetic biology applications under bioprocessing conditions. For example, it opens the possibility to explore further combinations of these promoters with other reporter genes, 5’UTR and RBS sequences [10, 11].

## Methods

### Strains

*Escherichia coli* strain BL21 (DE3) was used as expression host. *E. coli* BL21 was transformed with each plasmid and conserved at −80 °C in a solution of 40% *v*/v glycerol.

### Parts synthesis and assembly

The globin promoters used correspond to the reported by Koskenkorva and co-workers [3]. The sequences were obtained from the NCBI database and are detailed, together with their accession number, in the Additional file 1. A ribosome binding site (RBS) (Shine-Dalgarno sequence) and a spacer region of 8 bases were added previous to the start codon. FbFP sequence was taken from Evocatal (Düsseldorf, Germany, Cat. No.: 2.1.030) and the *rrnb* T1 terminator was added downstream. All the sequences were flanked by a HindIII restriction sequence and cloned in the same orientation (5′-3′). The complete sequences were synthesized and cloned in pUC57kan by GenScript (Piscataway, NJ, USA).

### Culture media

Precultures were grown in terrific broth (TB) consisting of 12 g L^−1^ tryptone, 24 g L^−1^ yeast extract, 12.54 g L^−1^, K_2_HPO_4_, 2.31 g L^−1^, KH_2_PO_4_, and 5 g L^−1^ glycerol. The main cultures were carried out using a mineral medium supplemented with 3-(N-morpholino)-propanesulfonic acid (MOPS) at a final concentration of 0.2 M, described elsewhere [2] and the pH was adjusted to 7.4 prior to sterilization. Glucose was added at final concentration of 5 g L^−1^. Kanamycin sulfate was used in all the cultures at a concentration of 50 μg mL^−1^.

### Culture conditions

For pre-culture development, 100 μL of cryopreserved cells were used to inoculate 10 mL of TB and grown at 30 °C in 250 mL Erlenmeyer flasks shaken at a frequency of 300 rpm with a shaking diameter of 50 mm for 8 h. 1 mL of this culture was transferred to 250 mL Erlenmeyer flasks containing 50 mL of the mineral medium. The cells were grown at 37 °C and shaking frequency of 300 rpm for 6–8 h. This time corresponds to the exponential growth phase, and the absorbance of the broth (measured at 600 nm) was around 2.0. This culture was used to inoculate the microbioreactors at an initial absorbance of 0.1 units. Microbioreactor cultures were performed using the BioLector system (m2p Labs, Beasweiler, Germany), which allows online measurement of cell growth, DOT, pH and fluorescence as indicator of FbFP expression using 48 round wells plates (MTP-R48-BOH, Lot 1402, m2p Labs, Beasweiler, Germany). Plates were sealed with a hydrophobic porous rayon sterile sealing film (AeraSeal, Excel Scientific, CA, USA). Cultures were performed at 37 °C, 85% humidity, shaking diameter of 3 mm, and shaking frequency of 700 rpm. Depending on the experiment, the culture volume per well was 1500 or 2400 μL. Biomass was monitored by scattered light (*λ*_*ex*_ = 620 nm; gain: 20). Fluorescence was used to monitor DOT (*λ*_*ex*_ = 520 nm; *λ*_*em*_ = 600 nm; gain: 83), pH (*λ*_*ex*_ = 485 nm; *λ*_*em*_ = 530 nm; gain: 45) and FbFP (*λ*_*ex*_ = 450 nm; *λ*_*em*_ = 492 nm; gain: 90). The OTR_max_ values were taken from Funke et al. 2009 [5]. All the experiments included three technical replicates.

### Data analysis

The initial data of scattered light and fluorescence intensity were subtracted from the measured data. Parameters for promoter characterization were determined during the aerobic or oxygen-limited phases. Specific fluorescence intensity was determined as the slope in the plot of fluorescence intensity (F-F_0_) versus scattered light intensity (I-I_0_) data points. The specific fluorescence emission rate was calculated as the product of *μ* multiplied by the specific fluorescence intensity. Fluorescence emission yields were calculated dividing the FbFP fluorescence intensity by the scattered light intensity of each time-point. For calculating the parameters under aerobic conditions, data corresponding to 2–4 h of culture were used for both OTR_max_ conditions, except for P_*vgb*_, for which data from 1 to 2.5 h (OTR_max_ ca. 7 mmol L^−1^ h^−1^) and 1–3.5 h (OTR_max_ ca. 11 mmol L^−1^ h^−1^) were used. For oxygen-limited conditions at OTR_max_ ca. 7 mmol L^−1^ h^−1^, the data from 4 to 7.5 (P_*kat*_), 4.9–8.7 (P_*Bs*_), 4–8.5 (P_*Cj*_), 4.2–8.9 (P_*Dr*_ and P_*Sc*_), 4.9–9.4 (P_*St*_) and 2.6–8.7 (P_*vgb*_) h of culture were used. For calculating the parameters in cultures under oxygen-limited conditions at OTR_max_ ca. 11 mmol L^−1^ h^−1^, the data from 4 to 7.5 (P_*kat*_), 4.9–8.7 (P_*Bs*_), 4.5–8.2 (P_*Cj*_), 5.2–8.9 (P_*Dr*_ and P_*Sc*_), 6.4–9.8 (P_*St*_) and 3.5–7.9 (P_*vgb*_) h of culture were used.

## Nomenclature

### Abbreviations

- ArcA Component A of the Aerobic respiratory control protein (the response regulator component)
- CRP Cyclic AMP receptor protein
- *d*_*0*_ Shaking diameter (mm)
- DOT Dissolved oxygen tension (% air saturation)
- FbFP FMN binding fluorescent protein
- FNR Fumarate and nitrate reductase (transcriptional activator)
- *n* shaking frequency (rpm)
- *q*_*F*_ Specific fluorescence intensity rate (AU AU^−1^ h^−1^)
- OTR Oxygen transfer rate (mmol L^−1^ h^−1^)
- V_L_ Volume of the liquid phase (μL)

### Symbols

- *λ*_*em*_ Emission wavelength [nm]
- *λ*_*ex*_ Excitation wavelength [nm]
- *μ* Specific growth rate (h^−1^)

## Additional file

Additional file 1: Complete sequences cloned in the plasmid pUC57kan used in this study. (DOCX 17 kb)
