# Supplementary material for: Evaluation of microbial globin promoters for oxygen-limited processes using Escherichia coli
Source: J Biol Eng. 2017 Nov 13;11:39. doi: 10.1186/s13036-017-0082-3 (PMC5682862; doi:10.1186/s13036-017-0082-3)
Supplement: Additional file 1: — Complete sequences cloned in the plasmid pUC57kan used in this study. (DOCX 17 kb) [file 13036_2017_82_MOESM1_ESM.docx]

**Supporting information**

**Evaluation of microbial globin promoters for oxygen-limited processes using *Escherichia coli***

Alvaro R. Lara,*^1^ Karim E. Jaén,^1^ Lars Regestein,^2^ Jochen Büchs**^2^

^1^Departamento de Procesos y Tecnología, Universidad Autónoma Metropolitana-Cuajimalpa. Av. Vasco de Quiroga 4871, Santa Fe, C.P. 05348 Mexico City, México

^2^RWTH Aachen University, AVT - Biochemical Engineering, Worringer Weg 1, 52074 Aachen, Germany

*Corresponding author. E-mail: [alara@correo.cua.uam.mx](mailto:alara@correo.cua.uam.mx)

**Corresponding author. E-mail: Jochen.Buechs@avt.rwth-aachen.de

**Promoters sequences used to control the expression of FbFP**

***Streptomyces coelicolor* A3(2) complete genome; segment 28/29**

**GenBank: AL939131.1**

**ACCESSION AL939131**

CATCACCCGCCTGCAGCACCTGGGTGTGGTGGAGGCGCGACGCGGTCGCGGCGGCGGGCTGACGCTGACC

GACCTGGGCCGGCGCGTCTCCGTGGGCTGGCTGGTGCGTGAACTCGAGGGCGAGGCCGAGGTGGTCGACT

GCGAGGGCGACAACCCCTGCCCGCTGCGCGGGGCCTGCCGGCTGCGGCGTGCGCTGCGCGACGCCCAGGA

GGCGTTCTACGCGGCACTCGACCCACTGACCGTGACCGACCTGGTGGCCGCACCGACCGGCCCGGTTCTG

CTCGGCCTGACGGACCGCCCCTCGGG

***Deinococcus radiodurans* R1 chromosome 2, complete sequence**

**NCBI Reference Sequence: NZ_CP015082.1**

**ACCESSION NZ_CP015082**

CCTTGAACAGGTAGGGCGGCGGCACCTCCGAGAGCCGGGCGAGTTCGGCGGCGCTCAGGGCGCGGCCCTG

CTCGGCCAGCATCACGGTGGCCCGCAGCGCGTATTCGGCGGTCTGTGAAAACATCCTCTTCTCCTCCCCT

CTGCCCACTGAATCTGGCCTGCCCACTGAATCTGGAGCTTGACATCCACTTTAGAGAGTCCTACCCTGAG

GCGTCAAACAAAGTGGATAAAAAGGTCCACTTTAAAGCCCCCTCTTCCCCGTCCTGAGGAGCCCCCCC

***Bacillus subtilis hmp* DNA for 7 ORFs, complete cds**

**GenBank: D78189.1**

**ACCESSION D78189**

TCAAATGGCAAGCCGGTGCCTGTACCGAGGGTTGTACCGGAAACAGAAGAAGAAATCATGCTGCATAATA

CAGCTGTACAGCGTGCTAATGAACGTAAGAACCGCAAGAGACACAGCCAGGCGCTTGCAAATGCGCTCGG

AACTGATAAGCCTTGGGTGAACGTTAAACCCAAAACATAAGTTGTTTTGGGTTTTTTTGTACTTTTCAAT

GGTGATTATTACAATGGTTAGTCCGTTTTTGCTAGGGGATTCTATAAAAAAGCAACAAATGTCATGTTAA

ATTGATAATTTTGTGACAACTTTATTAAAGATTCATTTTAGATATATCTTTTATTCGTAAGATCATGTAT

TTTAAAGATATATTTTAAATACATCTTTTCGAAAGGATTGTTTATAAA

***Campylobacter jejuni subsp. jejuni* NCTC 11168 complete genome; segment 6/6**

**GenBank: AL111168.1**

**ACCESSION AL111168**

CTTTAAAATTTGCCATAGTCCCTCTTTTTAATTTATTATCAATTATATAATTTAATAATTTAAAGATAATTTTAAATTTATATTTTTGAGCATAATTTTAACACAAGTCAATTTTTTTCTCCTTTTTAAGATATAAAATATCTCTTTTACAACAAAAAGGAGAAACT

***Salmonella enterica* subsp. enterica serovar Typhi str. CT18**

**NCBI Reference Sequence: NC_003198.1**

**ACCESSION NC_003198**

GATAACAGGTCTTGACAAAGGTTTTTACGCAAACGATTACCTATGCGTCAGATAAGGGTTTCCTGAACGAGAGCCTGACGAATTTCAACGGATTTCTTTTCAGCTTTGTGATGCAGATTTTTCACGTTGTTACCTCCATAACGTAAAGCAGAGAAGATCCATTTACAATGCAAGGGTATTTTTATAAGATGCATTTGATATACATTATTAGATTTTCACATAAAGGAAGCACGT
